# *Lactobacillus rhamnosus GG* Protects against Non-Alcoholic Fatty Liver Disease in Mice

**DOI:** 10.1371/journal.pone.0080169

**Published:** 2014-01-27

**Authors:** Yvonne Ritze, Gyöngyi Bárdos, Anke Claus, Veronika Ehrmann, Ina Bergheim, Andreas Schwiertz, Stephan C. Bischoff

**Affiliations:** 1 Department of Nutritional Medicine, University of Hohenheim, Stuttgart, Germany; 2 Department of Nutritional Science, Friedrich-Schiller-University, Jena, Germany; 3 MVZ Institute of Microecology, Herborn, Germany; INRA, France

## Abstract

**Objective:**

Experimental evidence revealed that obesity-associated non-alcoholic fatty liver disease (NAFLD) is linked to changes in intestinal permeability and translocation of bacterial products to the liver. Hitherto, no reliable therapy is available except for weight reduction. Within this study, we examined the possible effect of the probiotic bacterial strain *Lactobacillus rhamnosus GG* (LGG) as protective agent against experimental NAFLD in a mouse model.

**Methods:**

Experimental NAFLD was induced by a high-fructose diet over eight weeks in C57BL/J6 mice. Fructose was administered via the drinking water containing 30% fructose with or without LGG at a concentration resulting in approximately 5×10^7^ colony forming units/g body weight. Mice were examined for changes in small intestinal microbiota, gut barrier function, lipopolysaccharide (LPS) concentrations in the portal vein, liver inflammation and fat accumulation in the liver.

**Results:**

LGG increased beneficial bacteria in the distal small intestine. Moreover, LGG reduced duodenal IκB protein levels and restored the duodenal tight junction protein concentration. Portal LPS (P≤0.05) was reduced and tended to attenuate TNF-α, IL-8R and IL-1β mRNA expression in the liver feeding a high-fructose diet supplemented with LGG. Furthermore liver fat accumulation and portal alanine-aminotransferase concentrations (P≤0.05) were attenuated in mice fed the high-fructose diet and LGG.

**Conclusions:**

We show for the first time that LGG protects mice from NAFLD induced by a high-fructose diet. The underlying mechanisms of protection likely involve an increase of beneficial bacteria, restoration of gut barrier function and subsequent attenuation of liver inflammation and steatosis.

## Introduction

Over the last decades, progress was made in understanding the relationship between non-alcoholic fatty liver disease (NAFLD) and the intestinal microbiota [1]–[3]. Two major risk factors for NAFLD have been clearly identified - obesity and diabetes - both associated with changes in the intestinal microbiota [4], and with small intestinal bacterial overgrowth [5]. Furthermore, intestinal bacteria and their products may injure the liver and cause systemic inflammation as confirmed repeatedly by various studies [6], [7]. Nevertheless, understanding how the microbiota contributes to the pathology of diet-induced NAFLD remains a major challenge [8].

In western societies the prevalence of NAFLD increased to 20–30% within the general population, in the last years [9], [10]. Patients with NAFLD are characterized by a high prevalence of obesity ranging from 30% to 100% [11]. Most interestingly, NAFLD seems to be a predictor of type 2 diabetes mellitus in obese individuals [12]. About 20% of patients with steatosis develop a non-alcoholic steatohepatitis that may lead to severe hepatic and systemic diseases as well as increased mortality [13].

The high prevalence of NAFLD in the western society is likely resulting from lifestyle changes and particular dietetic behaviors. The latter may result in an increased energy intake, e.g. high amounts of potentially harmful food components such as sugars and fatty acids thought to promote metabolic syndrome, obesity and NAFLD [14]. In the last years it became clear that an inadequate energy intake which leads to obesity has implications on the gut microbiome [15]–[17]. Yet, it is unknown, if changes within the intestinal microbiota, which have been reported under high-fructose diet [18] may be related to the pathogenesis of liver steatosis.

In recent years, it became evident, that low grade inflammation due to metabolic endotoxemia has an implication on various diseases [19]. High fructose intake may lead to changes in the intestinal microbiome and intestinal barrier thus resulting in increased bacterial derived lipopolisaccharides, which are implicated in metabolic endotoxemia [19], [20].

Recently, probiotics conferring health benefits, e.g. by manipulation of the intestinal microbiota or by affecting the host, have been proven to ameliorate metabolic and infectious diseases [21], [22]. In particular, various probiotic lactobacilli strains promote beneficial effects, likely by anti-inflammatory actions and by stabilization of the intestinal barrier attenuating liver pathologies [23]–[25]. Most studies focused on a particular lactobacillus strain, *Lactobacillus rhamnosus GG* (LGG) and its anti-inflammatory mechanisms of action *in vitro* [25]–[27]. LGG is also known to prevent intestinal barrier impairment caused by inflammatory reactions and to reduce intestinal infection and diarrhea [26], [28]–[30].

In the here presented study, we examined, whether treatment with LGG may ameliorate experimental NAFLD induced by a high-fructose diet. We selected this NAFLD model, because we know from our previous experiments that the high-fructose diet induces not only NAFLD but also intestinal barrier impairment, portal lipopolysaccharide (LPS) elevation and lipid accumulation in the liver [14], [31]. Our results clearly show that LGG improves experimentally induced NAFLD *in vivo*. LGG modulates the small intestinal microbiome, restores small intestinal barrier impairment, and impairs genes involved in hepatic inflammation and lipid metabolism in our NAFLD model.

## Materials and Methods

### Mice and treatments

Mice were housed in a pathogen-free barrier facility accredited by the Association for Assessment and Accreditation for Laboratory Animal Care International (AAALAC). The local Institutional Animal Care and Use Committee (Regional Council Stuttgart, permit number: V 257/09 EM) approved all procedures. 6 weeks old female C57BL/6 mice (Janvier, Saint Berthevin Cedex, France) had for 8 weeks ad libitum access to water and MZ-diet (Sniff, Soest, Germany), 30% fructose solution (volume %) with enriched MZ-diet due to reduced food uptake, LGG about 5.2*10^7^ colony-forming units (cfu) per g body weight daily (InfectoPharm, Heppenheim, Germany) in water and MZ-diet, as well as 30% fructose solution with LGG in water and enriched MZ-diet (4 groups, n = 6). We used about 5*10^7^ cfu per g body weight since previous dose-response studies showed a protection of the intestine using LGG at 1–10^7^ cfu per g body weight [32]. Diet and body weight were assessed weekly, fluid intake every other day. After 8 weeks, mice were anesthetized via i.p. administration (ketamine at 80 mg/kg and xylazin at 6 mg/kg body weight). Blood was collected from the portal vein prior to killing. Specimen of small intestinal, and liver tissue were frozen immediately in liquid nitrogen for bacterial DNA, RNA and protein extraction. Portions of liver tissue were frozen-fixed in Tissue Tek® O.C.T.™ compound (Sakura Finetek Europe, AV Alphen aan den Rijn, Netherlands) or formalin-fixed and paraffin-embedded for subsequent sectioning and mounting on microscope slides.

### Alanine-aminotransferase activity

Portal plasma alanine-aminotransferase (ALT) activity (U/ml) was measured using a commercially available kit following the instructions of the manufacturer (Randox, Krefeld, Germany).

### Hepatic lipid analysis

Liver tissue pieces (50–100 mg) were homogenized in ice-cold 2× PBS and lipids were extracted. Triglycerides were assessed with a kit (Randox, Krefeld, Germany). Values were normalized to protein concentration, determined by Bradford assay, in liver homogenates (Bio-Rad Laboratories, Munich, Germany).

To determine hepatic lipid accumulation, liver sections were stained with Oil Red O and counterstained with hematoxylin or stained with hematoxylin and eosin (Sigma, Steinheim, Germany). Representative photomicrographs were captured at a 400× magnification using Axio Vert 200M (Zeiss, Jena, Germany).

### RNA isolation and real-time RT-PCR

Total RNA was extracted from liver and proximal small intestinal tissue samples using TriFast™ reagent (PEQLAB, Erlangen, Germany). RNA concentrations were determined spectrophotometrically, and 0.25 µg total RNA was reverse transcribed using an iScript DNA synthesis kit (BioRad Laboratories, Munich, Germany) followed by a DNAse digestion step (Fermentas, St. Leon Rot, Germany). PCR primers were designed using Primer3 software (Table 1). SsoFast EvaGreen Supermix (BioRad Laboratories, Munich, Germany) was used to prepare the PCR mix. The amplification reactions were carried out in an iCycler (BioRad Laboratories, Munich, Germany) with 40 cycles of a two-step PCR (denaturation 95°C for 35 s, denaturation 95°C for 5 s, annealing/extension 60°C for 10 s). The fluorescence intensity of each sample was measured at each temperature change to monitor amplification of the target gene. The comparative CT-method was used to determine the amount of target gene, normalized to an endogenous reference gene (18S) and relative to a calibrator (2^−ΔΔCt^). The purity of PCR products was verified by melting curves and gel electrophoresis.

**Table 1:** Primers used for mRNA detection.

	Forward (5′-3′)	Reverse (5′-3′)
**ChREBP**	CCACAGCGGACACTTCATGG	AGGCTCTCCAGATGGCGTTG
**ACC1**	CTTCCTCCTGATGAGCAACTCT	CGTGAGTTTTCCCAAAATAAG
**FAS**	TCTGGGCCAACCTCATTGGT	GAAGCTGGGGGTCCATTGTG
**TNF-α**	TGTCCATTCCTGAGTTCTG	GGAGGCAACAAGGTAGAG
**IL-1β**	CTTCAGGCAGGCAGTATC	CAGCAGGTTATCATCATCATC
**hIL-1β**	ATCTCCGACCACCACTAC	CACCACTTGTTGCTCCAT
**IL-8R**	GATCTGCCTCTACCCATGCAGAACA	TCCTGTGTGAGGGGACTCTGGT

TNF-α, tumor necrosis factor alpha; IL-1β, interleukin 1 beta; h, human; IL-8R, interleukin 8 receptor; ChREBP, carbohydrate response-element binding protein; ACC1, acetyl-CoA carboxylase 1; FAS, fatty acid synthase.

### Collection and preparation of small intestinal samples for analysis

Small intestinal tissue was frozen immediately in liquid nitrogen. Total bacterial DNA was isolated from the proximal and distal small intestine using a commercially available kit (QIAamp DNA Stool Mini Kit; Qiagen, Hilden, Germany).

### qPCR primers and conditions

Primers were selected to recognize the main bacterial phyla (Bacteroidetes, Firmicutes), the enterobacteriaceae family as representative of lipopolysaccharide (LPS) bearers, the lactobacilli group and LGG, respectively. The 16s rRNA gene DNA primers for the phyla Bacteroidetes and Firmicutes used in this study were designed by Baccetti De Gregoris et al. [33]. The primer for the *Lactobacilli/Enterococci* was designed by Schwiertz et al. [34]. The species specific primer for LGG was designed by Brandt and Alatossava [35].

PCR amplification and detection was performed using an ABI PRISM 7900HT sequence detection system (Applied Biosystems, Darmstadt, Germany) in optical-grade 96-well plates sealed with optical sealing tape. Each reaction mixture (25 µl) was composed of 12.5 µl of QuantiTect SYBR Green PCR Master Mix (Qiagen, Hilden, Germany), 2 µl primer mix (10 pmol/µl each), 9 µl sterile distilled H_2_O, and 1.5 µl stool DNA (10 ng/µl). For the negative control, 2 µl of sterile distilled H_2_O was added to the reaction solution instead of the template DNA solution. A standard curve was produced using the appropriate reference organism to quantify the qPCR values into number of bacteria/g. The standard curves were prepared in the same PCR assay as for the samples. The fluorescent products were detected in the last step of each cycle. A melting curve analysis was carried out following amplification to distinguish the targeted PCR product from the non-targeted PCR product. The melting curves were obtained by slow heating at temperatures from 55 to 95°C at a rate of 0.2°C/s, with continuous fluorescence collection. The data was analyzed using the ABI Prism software. The real-time PCRs were performed in triplicate, and average values were used for enumeration.

### Protein expression

To prepare total tissue protein, snap-frozen small intestine samples were homogenized in a lysis buffer (20 mM MOPS, 150 mM NaCl, 1 mM EDTA, 1% Nonidet P-40, 1% sodium deoxycholate, 0.1% SDS) containing a protease inhibitor mix (Roche, Mannheim, Germany). Protein lysates (30 µg protein per well) were separated in a 10% SDS-polyacrylamide gel and transferred to Hybond™-P polyvinylidene difluoride membranes. Blots were then probed with antibodies against occludin (1∶500, in 5% skim milk overnight; Zymed, Wien, Austria), claudin-1 (1∶500, in 5% skim milk overnight; Invitrogen, Darmstadt, Germany), IκB/pIκB kinase (1∶1000 in 5%, bovine serum albumin, Cell Signaling, Danvers, MA), or ZO-1/2 (1∶250, in 5% skim milk overnight; Invitrogen, Darmstadt, Germany), respectively. The bands were visualized using Super Signal Western Dura kit (Pierce, Perbio Science, Rockford, IL). To ensure equal loading, all blots were stained with Ponceau red; signals were normalized to β-actin (1∶750 in 2.5% bovine serum albumin overnight; New England Biolabs, Frankfurt, Germany). Protein bands were analyzed by densitometry using the Flurochem Software AlphaEaseFS (Alpha InnoTec, Kasendorf, Germany).

### Lipopolysaccharides assay

Portal plasma samples were heated at 73°C for 20 min. Lipopolysaccharides (LPS) concentration was determined using a limulus amebocyte lysate assay kit (concentration range of 0.015–1.2 EU/mL; Charles River, Wilmington, MA).

### Human epithelial cell culture

The human epithelial cell line caco2 (carcinoma colon-2 cell, American Type Culture Collection, Manassas, VA) is not only accepted as a model for intestinal barrier [36] but for fructose metabolism [37] as well. Caco2 cells, passages 37–47, were maintained in Dulbecco's modified Eagles' medium high glucose (4,5 g/l, PAA Laboratories, Pasching, Austria) supplemented with 20% fetal bovine serum (FBS) (Biochrom AG, Berlin Germany), 1% non-essential amino acids (Biochrom AG, Berlin Germany) and 1% penicillin-streptomycin (PAA Laboratories, Pasching, Austria) in a humidified 5% CO_2_ atmosphere at 37°. Cells were fed every 2 or 3 days and transferred after reaching 75% of confluence to transwell systems (transparent PET Membrane, 0.4 µm pore size, 4.2 cm^2^ growth area; BD Falcon, Heidelberg, Germany) at a density of 5*10^5^ cells per well.

After 13 days cells differentiated completely and were maintained with DMEM supplemented with 1% NEA and 1% penicillin-streptomycin, without FBS 24 h.

### Transepithelial electrical resistance and dextran permeability as measurements of barrier function

The transepithelial electrical resistance (TER) was measured with a Millicell ERS voltmeter (Millipore, Bedford, MA). In addition the transepithelial flux of a 4-kDa fluorescein isothiocyanate (FITC)-labeled dextran molecule (Sigma Aldrich, Steinheim, Germany) was detected. In brief, 24 hours before the experiment started, FITC-labeled dextran was added to the apical side of the monolayers with a final concentration of 0.2 mg/ml. After incubation of 0, 3 and 23 hours 100 µl aliquots of the basolateral medium were removed and FITC-dextran fluorescence was measured (Photometer Synergy HT; Bio-Tek, Winoosky, VT).

### Caco2 treatment with fructose and *Lactobacillus rhamnosus GG*

Before the treatment caco2 monolayers were washed twice with PBS, to remove the penicillin-streptomycin and the FITC-dextran. Caco2 cells were treated with either conditioning medium (DMEM with 1% NEA) (control group), 25 mM fructose in DMEM (fructose group), 5*10^7^ cfu per well LGG (LGG group) or 25 mM fructose and 5*10^7^ cfu per well LGG (fructose+LGG group). The fructose solution was filtered sterile before using.

LGG (Infectopharm, Heppenheim, Germany) was isolated, purified and cultured in MRS medium under anaerobic conditions at 37°C and 5% CO_2_. A preparatory culture of LGG was prepared two days before the experiment, 26 hours before the experiment the main culture of LGG was prepared. At the same time a serial dilution was dispersed on agar and cultured under anaerobic conditions. Directly before monolayers were treated with LGG, the optical density of the culture was measured and the LGG colonies on the Agar-plates were counted. The calculated amount of LGG cfu was washed with PBS and resuspended in medium with LGG 5*10^7^ cfu per well at 37°C and 5% CO_2_ for 24 hours.

### Immunohistologically staining of tight junctions

Squares of caco2 cell monolayers were cut, placed on slides, fixed (4% paraformaldehyde, 15 min) and washed (1×PBS with 0,5% Triton-X-100). At room temperature preparations were blocked (wash buffer with 5% normal goat serum, 30 min) and primary antibody was added (occludin, mouse 1∶100 or claudin-1 mouse 1∶100; Invitrogen, Darmstadt, Germany) for one hour. After washing (1×PBS, 5 min) secondary antibody in blocking solution was added (goat anti-mouse Cy3 1∶500, Millipore, Bedford, MA) and incubated 1 hour in the dark. DAPI staining (1×PBS, 1∶4000, 5 min) was used to stain nuclei. Preparations were washed 5 min and mounted (Vectashield, Burlingame, CA). Representative photomicrographs were captured at a 630× magnification using Axio Vert 200M (Zeiss, Jena, Germany).

### Statistical analyses

All results are presented as means ± SEM. One-way ANOVA analysis with Tukey's post hoc test and Bartlett's test for equal variances was used. If the Bartlett's test showed no equal variances, the Kruskal Wallis test with Dunn's post hoc test was applied. A *P* value<0.05 was determined as the level of significance prior to study start. The software GraphPad Prism 5 (GraphPad Software, La Jolla, CA) was used for calculation and graph design.

## Results

### Nutritional and weight parameters in the four feeding groups

We investigated the role of the probiotic LGG on the development of NAFLD in mice fed a control diet or a high-fructose diet regarding total caloric intake from food and fructose intake. The total caloric intake was increased in the high-fructose groups compared to the control groups independent of whether animals received LGG or not (Table 2). As reported earlier by our group [31], we could show that a high-fructose diet does not cause a significant increase in body weight. Similarly, LGG supplementation did not influence body weight (Table 2). Nevertheless, elevated ALT concentration in plasma was almost normalized by LGG in high-fructose fed mice (Table 2).

**Table 2:** Nutritional and weight parameters.

	C	F	CLGG	FLGG
***n***	6	6	6	6
**Total caloric intake [kcal/g body weight/wk]**	3.61±0.2	4.29±0.1^a^	4.15±0.2	4.37±0.1^a^
**Fructose intake [kcal/g body weight/wk]**		2.3±0.1		2.6±0.1
**Weight gain [g]**	4.4±0.5	5.3±0.6	3.6±0.8	4.4±0.6
**ALT [U/ml]**	2.8±0.7	6.7±1.4^a^	2.8±0.8	2.5±0.6^b^

Animal groups: C, control diet; F, high-fructose diet; CLGG control diet supplemented with *Lactobacillus rhamnosus GG* (LGG); FLGG, F supplemented with LGG; ALT, alanine-aminotransferase. The detailed feeding protocols of the four animal groups are described in material and methods. Data are shown as means ± SEM (*n* = 6).

a P<0.05 compared to C;

b P<0.05 compared to F.

### *Lactobacillus rhamnosus GG* ameliorated fat accumulation in the liver

**Figure 1:**
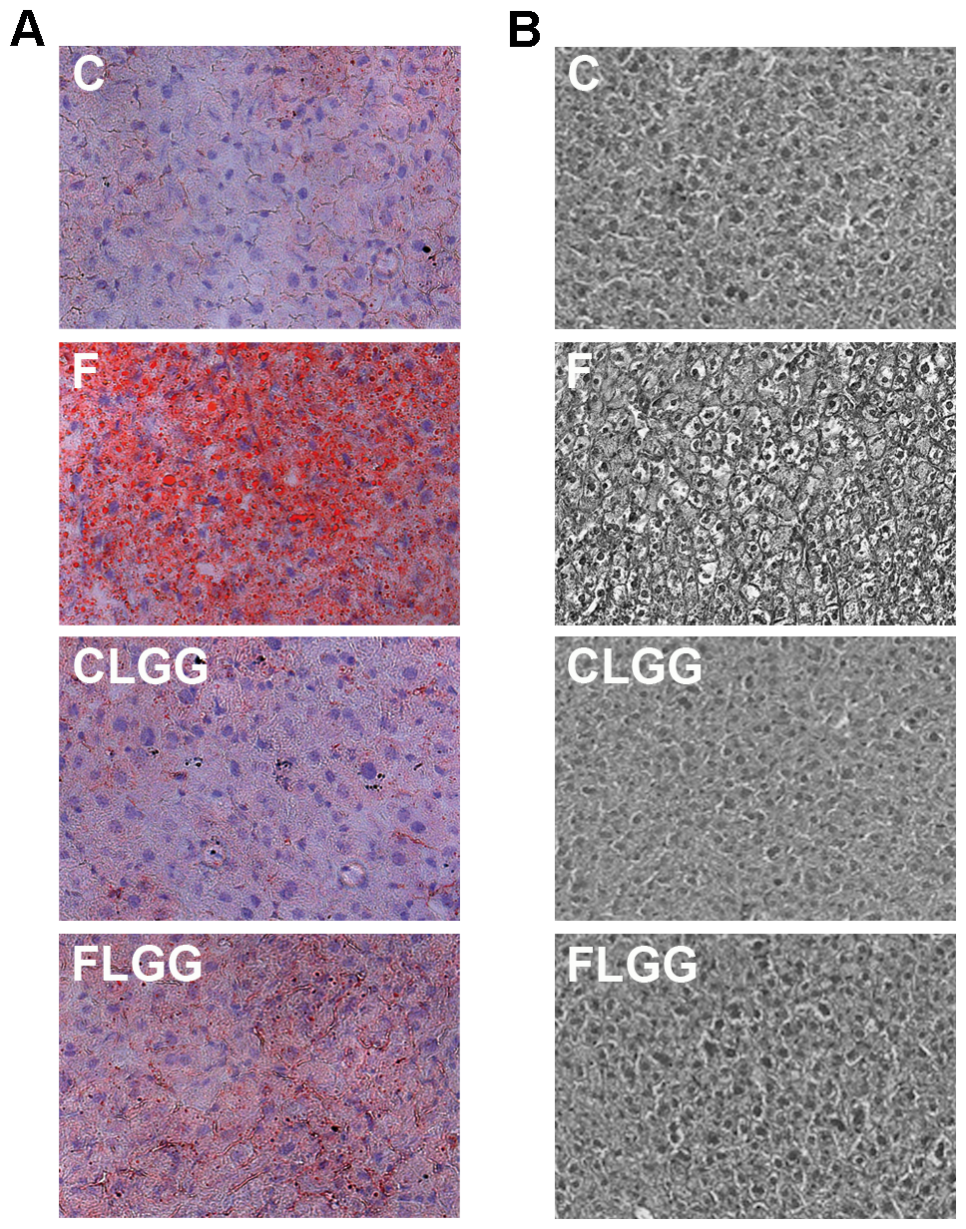
*Lactobacillus rhamnosus GG* ameliorates fructose-induced hepatic fatty acid accumulation. Representative Oil-Red-O (A) and hematoxilin & eosin (B) stainings showing fat accumulation in the liver. Abbreviations: C, control diet; F, high-fructose diet; CLGG, control diet with *Lactobacillus rhamnosus GG* (LGG) supplementation; FLGG, F with LGG supplementation.

Although high-fructose diet does not cause significant weight gain, we know from our previous experiments that fructose induces substantial steatosis [20]. Therefore, we were interested, if LGG affects hepatic fat accumulation in our mouse model. Representative histochemical stainings showed that over all liver fat accumulation was strongly reduced by LGG in the high-fructose diet fed mice (Fig. 1A). In addition, liver histology of the fructose fed group clearly showed hepatocellular ballooning cells known for a higher degree in steatosis in contrast to the almost normalized liver histology of LGG and fructose fed mice (Fig. 1B).

### Hepatic expression of genes involved in lipid metabolism

**Figure 2:**
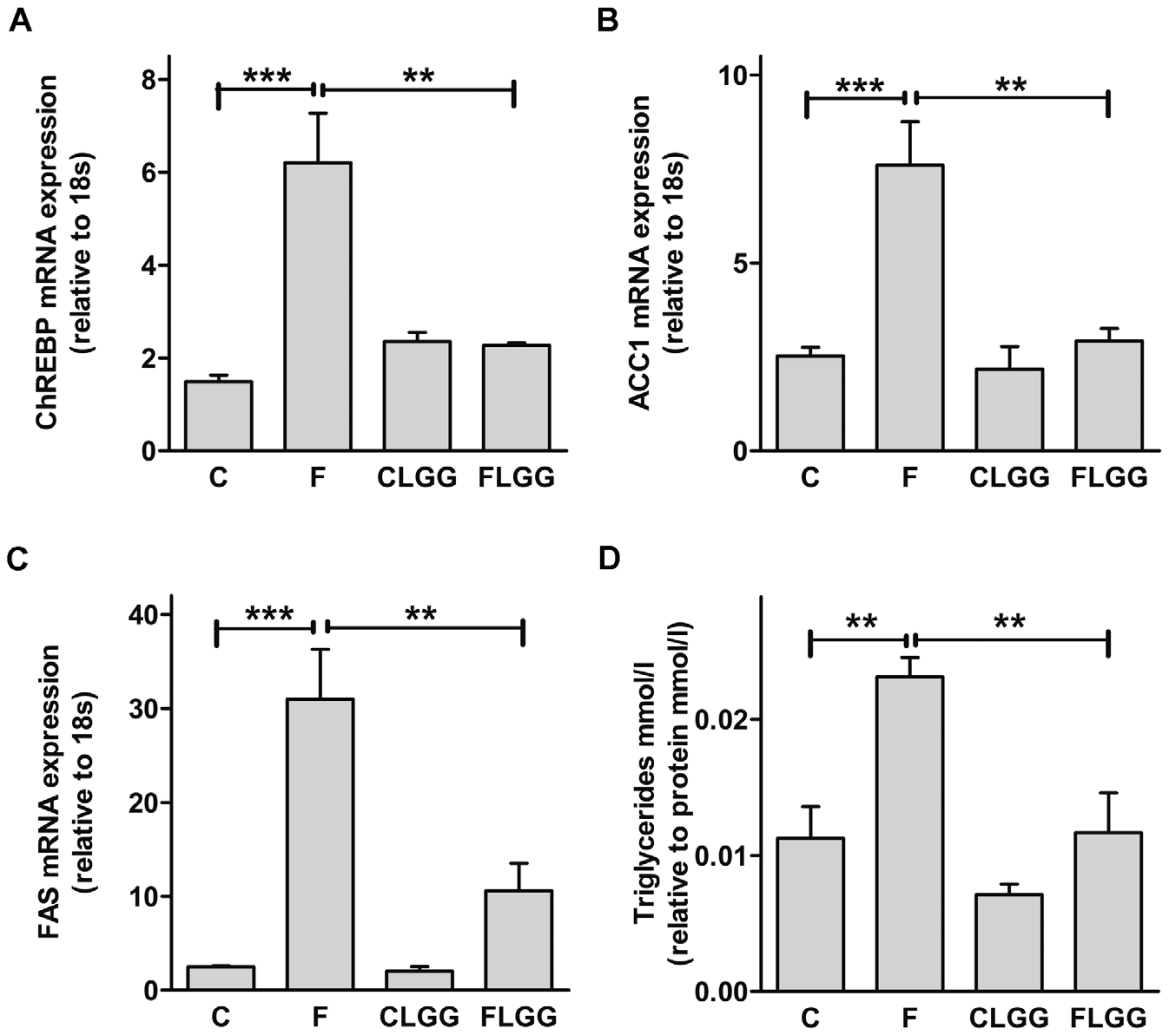
*Lactobacillus rhamnosus GG* modulates fructose-induced hepatic fatty acid metabolism. Hepatic ChREBP (A), ACC1 (B) and FAS (C) mRNA expression was measured. Concentrations of triglycerides in the liver were analysed (D). Data are shown as means ± SEM (**P<0.01; ***P<0.001; *n* = 4–6). Abbreviations: see Figure 1; ChREBP, carbohydrate response element-binding protein; ACC1, acetyl-CoA carboxylase 1; FAS, fatty acid synthase.

We measured the transcription factor carbohydrate-responsive element-binding protein (ChREBP) [38]. In addition, since ChREBP is required for glucose-induced expression of the lipogenic genes acetyl-CoA carboxylase 1 (ACC1) and fatty acid synthase (FAS) [38] we investigated, if their expression is also affected by LGG treatment feeding a fructose-rich diet. We found an increased expression of ChREBP, ACC1 and FAS feeding the fructose rich diet that was significantly reduced after LGG supplementation (Fig. 2A,B,C). In addition, LGG almost normalized elevated hepatic triglyceride concentration in high-fructose fed mice (Fig. 2D).

### *Lactobacillus rhamnosus GG* reduced liver inflammation

**Figure 3:**
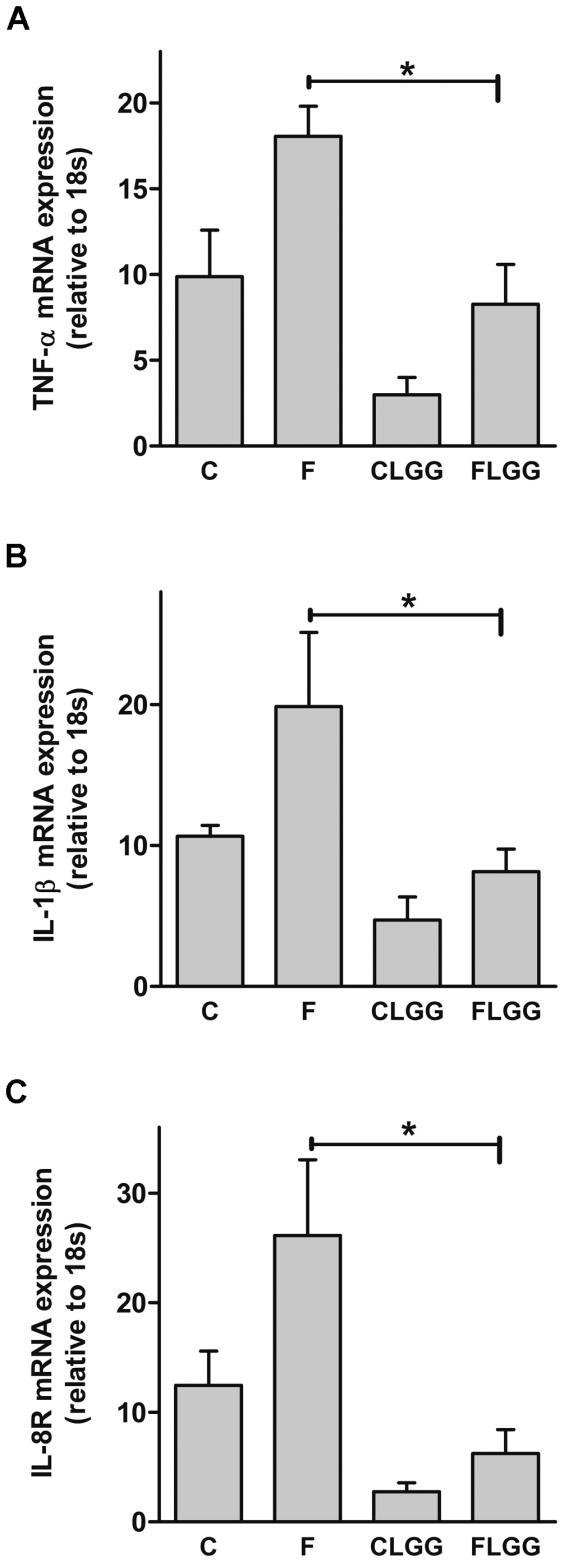
*Lactobacillus rhamnosus GG* ameliorates fructose-induced liver inflammation. Hepatic TNF-α (A), IL-1β (B) and IL-8R (C) mRNA expression was measured. Data are shown as means ± SEM (*P<0.05; *n* = 4–6). Abbreviations: see Figure 1; TNF-α, tumor necorsis factor alpha; IL-1β, interleukin 1 beta; IL-8R, interleukin 8 receptor.

We investigated inflammatory markers previously shown to be modulated by LGG treatment [26], [27], [39], [40] in the liver. We observed that the mRNA concentrations encoding for the two pro-inflammatory cytokines (TNF-α, IL-1β) and the cytokine receptors (IL-8R), respectively, were reduced (P<0.05) in LGG and fructose-treated animals compared to high-fructose fed mice (Fig. 3).

### *Lactobacillus rhamnosus GG* improved markers of intestinal barrier function

**Figure 4:**
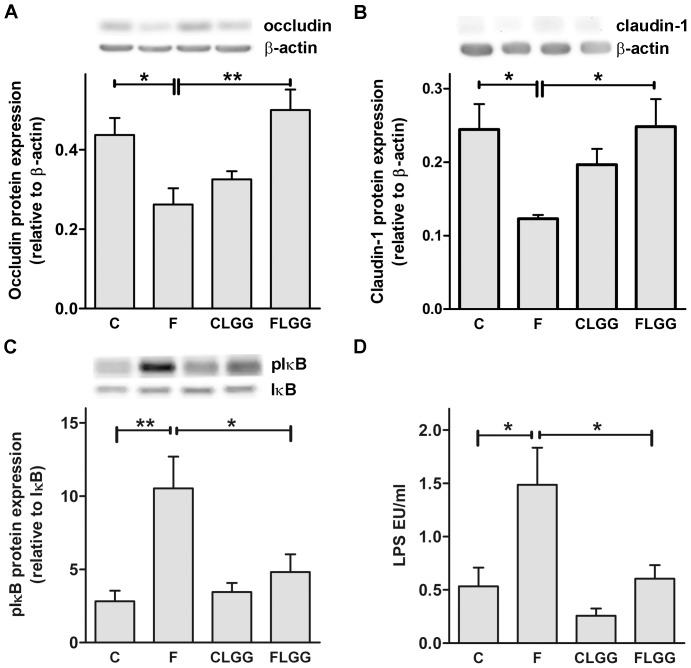
*Lactobacillus rhamnosus GG* improves markers of intestinal barrier function. The tight junction molecules occludin (A), and claudin-1 (B), as well as the inflammatory marker pIκB kinase (C) were analysed in the proximal intestine. Representative western blots and quantitative analyses of the blots are shown (A–C). LPS in portal plasma was measured (D). Data are shown as means ± SEM (*P<0.05, **P<0.01; *n* = 4–6). Abbreviations: see Figure 1; LPS; lipopolysaccharides.

Previous studies provided evidence for enhanced LPS levels in the portal vein following high-fructose diet, and for LPS translocation being one trigger for liver inflammation occurring in this animal model [20], [31], [41]. To determine whether changes in portal LPS levels and intestinal inflammation could be associated with the intestinal barrier, we measured the tight junction proteins occludin and claudin-1 (Fig. 2A,B). Occludin and claudin-1 protein expression was significantly reduced in mice fed high-fructose diet compared to control diet. This reduction was removed following oral treatment of the mice with LGG (Fig. 2C,D). In contrast, zonula occludens 1 and 2 protein expression was neither influenced by high-fructose diet nor LGG treatment (data not shown). Furthermore, the duodenal protein expression of the inflammatory marker IκB increased substantially in high-fructose diet fed mice compared to control mice and was almost normalized in LGG-treated fructose fed mice (Fig. 4C). In addition, we measured almost tripled portal LPS concentrations in mice fed high-fructose diet. Most interestingly, oral treatment with LGG almost normalized the elevated portal LPS levels in high-fructose diet fed mice (Fig. 4D).

To further substantiate if the barrier impairment is indeed caused by fructose, we performed *in vitro* studies using an established human epithelial cell culture model (caco2). We added either fructose, or LGG, or fructose and LGG to the cell culture and measured tight junction protein expression as well as IL-1β mRNA expression as a marker of inflammation. We saw neither a significant reduction of occludin or claudin-1 protein expression or an increase in inflammation in caco2 cells following fructose application. Thus, there was no normalization of the expression of these tight junctions or the inflammatory marker IL-1β following LGG treatment (Figure S1).

### Effect of *Lactobacillus rhamnosus GG* on the small intestinal microbiota

**Figure 5:**
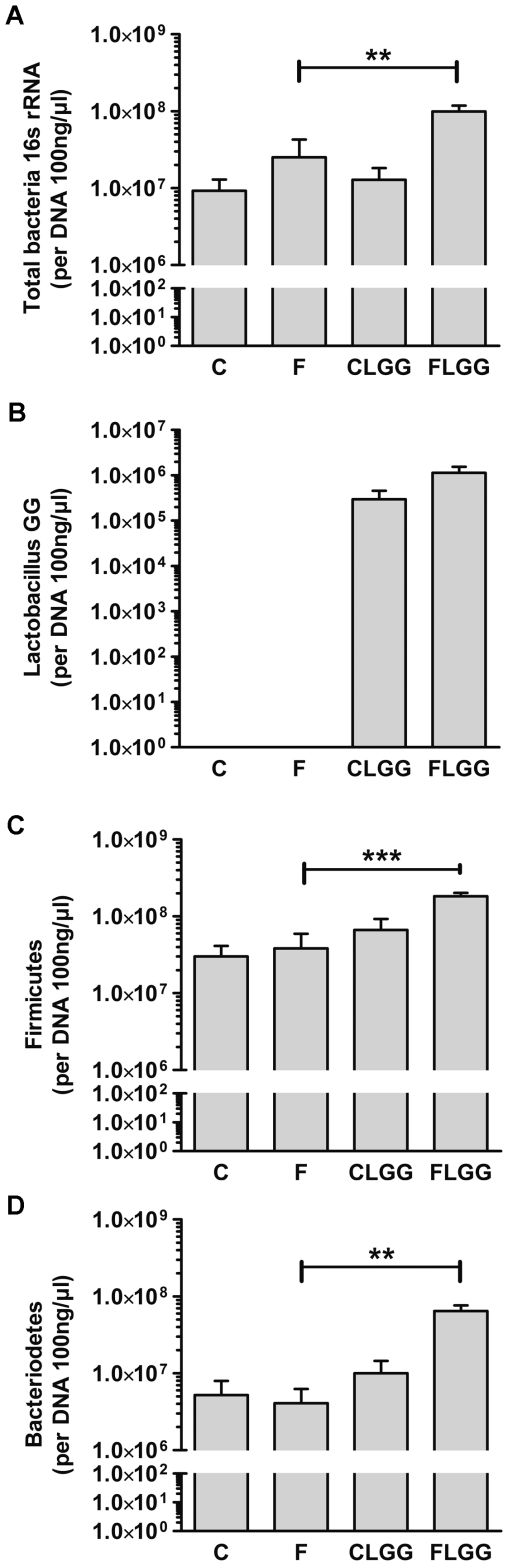
*Lactobacillus rhamnosus GG* elevates bacterial numbers in the distal small intestine. Total bacterial numbers (A), LGG numbers (B), the phyla Firmicutes (C), and Bacteriodetes (D) were measured via qPCR. Data are shown as means ± SEM (**P<0.01, ***P<0.001; *n* = 5–6). Abbreviations: see Figure 1.

Recently, a report showed that LGG alters the total number of bacteria and the ratio of particular microbial groups such as Firmicutes and Bacteroidetes in the small intestine, but not in the feces of mice [42]. Therefore, we analyzed diverse phyla of the murine small intestinal microbiome using qPCR. Our data indicated that proximal small intestinal microbiota was not influenced by LGG (data not shown). However, we observed an increase in the microbiota in total bacterial numbers including the phyla Firmicutes and Bacteroidetes in the distal small intestine following a high-fructose diet and LGG compared to fructose fed mice (Fig. 5). However, numbers of total *Lactobacilli/Enterococci* were not influenced by LGG (data not shown).

## Discussion

We show that the probiotic LGG, administered orally, attenuates the development of high-fructose induced NAFLD. This finding is substantiated at different levels including the composition of the small intestinal microbiota, the gut barrier function, the concentration of portal lipopolysaccharides (LPS), liver inflammation and hepatic fat accumulation.

In most studies, the protective effect of LGG against inflammatory reactions was analyzed *in vitro* using cell culture such as the caco2 cell line [26]–[28], [43]. In the few hitherto performed *in vivo* studies with LGG, a high-fat diet or ethanol was administered instead of a high-fructose diet [25], [44]. Here, we measured LGG effects *in vivo* in mice and *in vitro* in caco2 cell culture applying high-fructose doses what leads to NAFLD in mice and to barrier impairment in caco2 cells [40].

So far, little is known about the impact of probiotic consumption on NAFLD [45]. Here, we show that the impact of LGG on the composition of the small intestinal microbiota seems to play a role for the prevention of NAFLD. This conclusion can be drawn from the fact that LGG induced an increase of the total numbers of the distal small intestinal microbiota and especially, a shift towards the beneficial bacteria phyla Firmicutes and Bacteroidetes. These results are in agreement with Ji et al. who reported a modulation in total bacterial number of the phyla Firmicutes and Bacteriodetes in the small intestine of mice following LGG application [42]. However, Ciorba et al. [32] did not find a shift in bacterial family composition following feeding LGG for three days by gavage. The beneficial effect of the increase in the two bacterial phyla may be due to the fact that members of the Firmicutes produce butyrate which is known to regulate gut barrier function [46]. The herein described effects of LGG may thus be indirect due to an attenuation of the altered barrier function caused by the high-fructose diet. Indeed, we found that the expression of two major tight junction proteins, occludin and claudin-1, are enhanced if LGG is administered to mice receiving a high-fructose diet. In addition, not only markers of intestinal barrier function, but also of intestinal inflammation, such as pIκB kinase expression, were normalized feeding LGG in combination with the high-fructose diet.

The beneficial effects of LGG on the intestinal barrier function possibly result in the here shown decreased translocation of LPS from the gut to the liver and thus a decreased liver inflammation and steatosis. Likely, not only steatosis but also liver injury is prevented, since LGG also reduced ALT activity in portal plasma in mice fed a high-fructose diet [44]. Interestingly, similar results were shown for the probioticum *Lactobaccilus casei shirota* [47]. Furthermore, a human study showed that a synbiotic, consisting of several pro- (4 non-urease producing bacteria: *Pediacoccus pentoseceus* 5–33:3, *Leuconostoc mesenteroides* 32–77:1, *Lactobacillus paracasei* 19, *Lactobacillus plantarum* 2592) and prebiotic (β-glucan, inulin, pectin, resistant starch) components, significantly improved serum ALT and LPS levels as well as signs of hepatic encephalopathy in 50% of patients with cirrhosis of different origin [48]. In contrast to our findings, the probiotic strain *Lactobacillus acidophilus* had no effect on intestinal permeability, but ameliorated high-fat induced NAFLD in rats [25]. This might be due to the fact that the microbiota was not influenced by *Lactobacillus acidophilus* and that the lactulose/mannitol test [49] was used to assess intestinal barrier function instead of tight junction protein expression and portal LPS quantification.

To further confirm our findings, we performed apart from our *in vivo* approach *in vitro* studies using a human epithelial line, because it has been shown that LGG improves epithelial cell barrier injury induced by bacterial infection [28]. We observed no significant enhancement of occludin and claudin-1 expression after LGG and fructose-administration compared to fructose treated cells. Our representative pictures show that LGG treatment might support the restoration of the tight junction network within the fructose-treated human epithelial cell monolayer. However, these findings need further confirmation.

As shown earlier, probiotics inhibit TNF-α inflammatory activity and improve NAFLD [50]. We underline these findings showing normalization of increased TNF-α, and in addition for the inflammatory markers IL-1β and IL-8R in the liver of high-fructose diet fed mice with LGG supplementation.

Hepatic fat metabolism also seems to be influenced by the presence of probiotics; although the mechanisms by which probiotic bacteria might act on the liver are still unclear [45]. ChREBP has an important role in hepatic *de novo* lipogenesis targeting genes involved in triglyceride synthesis e.g. ACC1 and FAS [38]. Interestingly, the high-fructose diet lead to an increase of these molecules, which were normalized following LGG supplement to the mice. A similar result was found by Ji et al.feeding LGG and NR28 to C57BL/6 mice [42].

**Figure 6:**
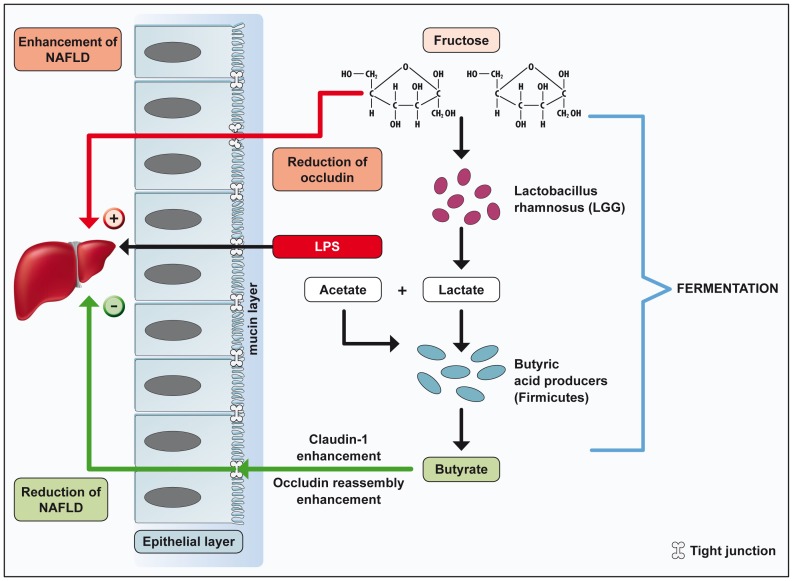
*Lactobacillus rhamnosus GG* improves diet-induced NAFLD. LGG ameliorates high-fructose diet-induced NAFLD via modulation of the intestinal microbiota. LGG products e.g. lactate may increase butyrate producing Firmicutes leading to an improved intestinal barrier and reduced portal plasma LPS concentrations as well as a decreased inflammation and fatty acid accumulation in the liver. Abbreviations: NAFLD, non-alcoholic fatty liver disease; LPS, lipopolysaccharides.

The mechanism of action of LGG in the present setting is unknown, as we know little about the probiotic mechanisms of actions in general [30]. To hypothesize on possible mechanisms of action, the pathomechanisms of liver steatosis induced by a high-fructose diet needs to be discussed. One most likely, although probably not the only, mechanisms of fructose-associated NAFLD is liver inflammation and damage induced by bacterial products derived from the intestine [31]. We and others provided evidence supporting the hypothesis that a high-fructose diet causes elevated LPS concentrations in the portal vein entering the liver and triggering for inflammatory reactions [18]. This finding requires that the translocation of LPS from the gut into the portal vein is enhanced by diet, and suggests that the intestinal barrier is altered. Indeed, we could confirm in previous as well as in the present study that markers of the intestinal barrier such as tight junction protein expression are altered following such a diet [20]. In this study, we postulate that bacterial products produced by the increased number of Firmicutes such as butyrate might improve the fructose-induced impairment of the intestinal barrier (Fig. 6).

In conclusion, we could show that LGG protects against diet-induced NAFLD using an established NAFLD mouse model. Underlying mechanisms involve a modulation of the small intestinal barrier which seems to be altered by high-fructose diet and partially restored by the LGG induced increase of butyrate producing bacteria (Fig. 6).

The question, whether the increase of the intestinal microbiota as a whole, or the composition of the intestinal microbiota plays a role for beneficial effects of LGG in our NAFLD model must be further evaluated.

## Supporting Information

Figure S1 **Effect of** ***Lactobacillus rhamnosus GG*** **on tight junction expression in human epithelial cells.** Immunohistological staining of occludin (green) and claudin-1 (red) of a human epithelial cell culture layer (630×, blue = cell nuclei) are shown (A). Representative western blots of occludin, claudin-1, and β-actin, respectively, as well as quantitative analyses of the blots (B,C) are shown. IL-1β mRNA expression (D) was measured. Data are shown as means ± SEM (*n* = 3). Abbreviations: see Figure 1; IL-1β, interleukin 1 beta.
(TIF)
