# Supplementary figures and images for: Lactobacillus rhamnosus GG Protects against Non-Alcoholic Fatty Liver Disease in Mice
Source: PLoS One. 2014 Jan 27;9(1):e80169. doi: 10.1371/journal.pone.0080169 (PMC3903470; doi:10.1371/journal.pone.0080169)

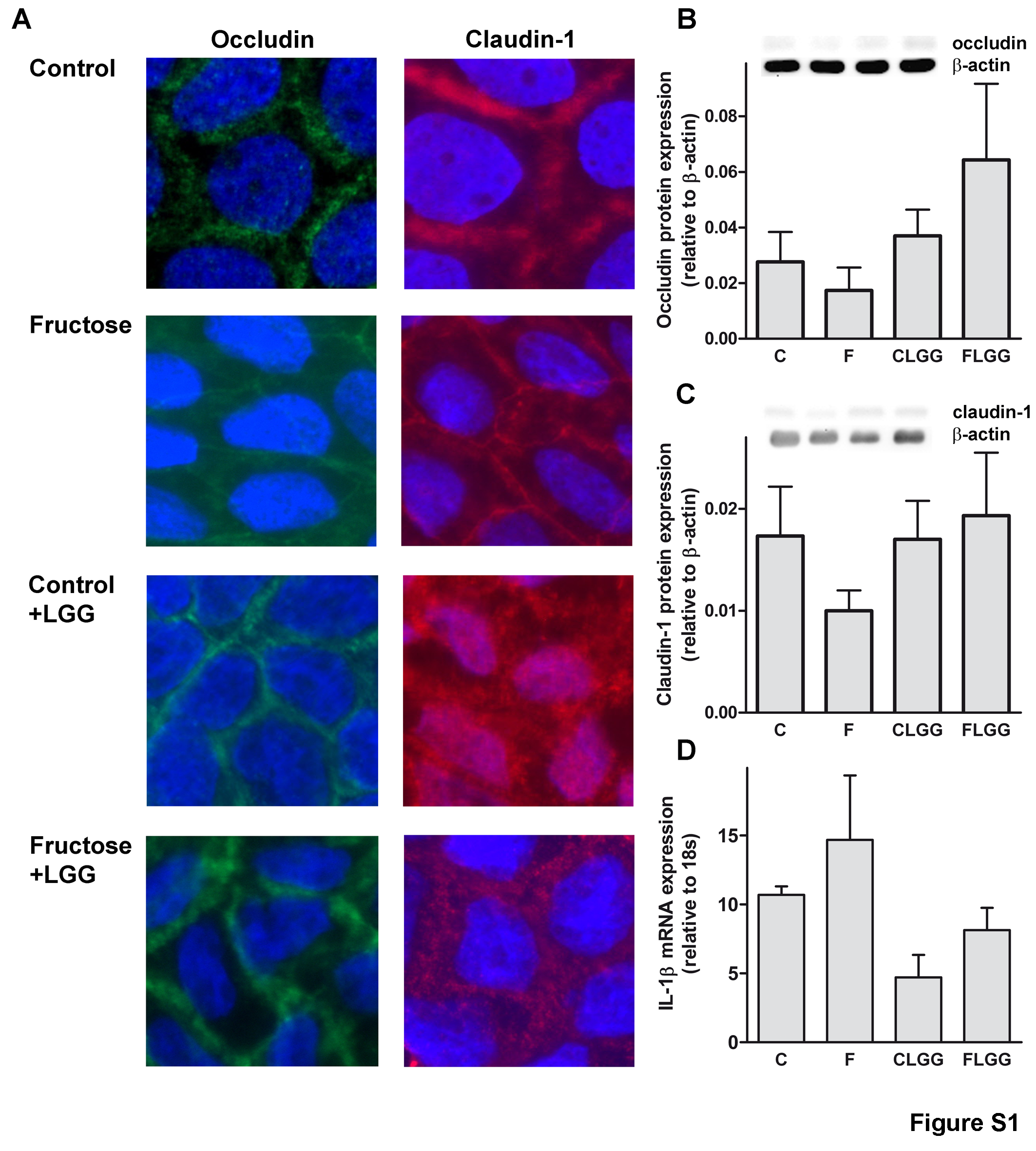

Supplement: Figure S1 — Effect of Lactobacillus rhamnosus GG on tight junction expression in human epithelial cells. Immunohistological staining of occludin (green) and claudin-1 (red) of a human epithelial cell culture layer (630×, blue = cell nuclei) are shown (A). Representative western blots of occludin, claudin-1, and β-actin, respectively, as well as quantitative analyses of the blots (B,C) are shown. IL-1β mRNA expression (D) was measured. Data are shown as means ± SEM (n = 3). Abbreviations: see Figure 1; IL-1β, interleukin 1 beta. (TIF) [file pone.0080169.s001.tif]
